# Methodological challenges in using the Finnish Hospital Discharge Register for studying fire-related injuries leading to inpatient care

**DOI:** 10.1186/1472-6947-13-36

**Published:** 2013-03-15

**Authors:** Kari Haikonen, Philippe Lunetta, Pirjo M Lillsunde, Reijo Sund

**Affiliations:** 1 Injury Prevention Unit, National Institute for Health and Welfare, Mannerheimintie 164a, P.O. Box 30, FI-00271 Helsinki, Finland; 2 Department of Forensic Medicine, University of Helsinki, Kytösuontie 11, P.O. Box 40, , Helsinki, FI-00014, Finland; 3 National Institute for Health and Welfare, Mannerheimintie 164a, P.O. Box 30, , Helsinki, FI-00271, Finland; 4 Service Systems Research Unit, National Institute for Health and Welfare, Nauvontie 4, P.O. Box 30, , Helsinki, FI-00271, Finland

**Keywords:** Discharge register, Injury, Fire, E-coding, Register study, Burns

## Abstract

**Background:**

The objective was to examine feasibility of using hospital discharge register data for studying fire-related injuries.

**Methods:**

The Finnish National Hospital Discharge Register (FHDR) was the database used to select relevant hospital discharge data to study usability and data quality issues. Patterns of E-coding were assessed, as well as prominent challenges in defining the incidence of injuries. Additionally, the issue of defining the relevant amount of hospital days accounted for in injury care was considered.

**Results:**

Directly after the introduction of the ICD-10 classification system, in 1996, the completeness of E-coding was found to be poor, but to have improved dramatically around 2000 and thereafter. The scale of the challenges to defining the incidence of injuries was found to be manageable. In counting the relevant hospital days, psychiatric and long-term care were found to be the obvious and possible sources of overestimation.

**Conclusions:**

The FHDR was found to be a feasible data source for studying fire-related injuries so long as potential challenges are acknowledged and taken into account. Hospital discharge data can be a unique and powerful means for injury research as issues of representativeness and coverage of traditional probability samples can frequently be completely avoided.

## Background

The number of lives lost due to fire-related injuries in relation to the population size of Finland is among the highest in the westernized countries [1]. The reasons and circumstances behind deaths due to fire have been studied exhaustively. It is well known that each fire-related injury represents a potential risk of fire-related death, and that burn injuries often cause long hospitalisations, leading to a high burden of injury in terms of costs and morbidity [2-6]. However, the actual fire-related injuries are a less studied topic.

The most comprehensive data source for nationwide assessment of severe fire-related injuries in Finland is the Finnish National Hospital Discharge register (FHDR). The FHDR is an important and widely used source for research data and official statistics in Finland. Hospital discharge data have also been successfully used for injury surveillance in other countries, although there have been certain recognised challenges [7-9].

For instance, it is typical of fire-related injuries (i.e. injuries caused by exposure to smoke, fire or flames) that these injuries require multiple admissions to hospital [10,11]. Although variables indicating whether the admission was urgent, elective or a transfer between hospitals may exist in hospital discharge registers, a specific variable explicitly indicating the first admission for the injury or condition is typically missing [12]. In addition, determining resource usage and consequences induced by the fire-related injury may be complicated. It is not sufficient to calculate the length of stay by using admission and discharge dates of a single care period. By counting only the inpatient days in the records with codes indicating fire-related injury, many complications arising as consequences or sequelae of the injury - including pneumonia, urinary tract infection, respiratory failure and septicemia [13,14] - could be erroneously excluded [15]. It is also possible that patients with psychiatric problems may self-inflict fire-related injuries during psychiatric hospitalisation or are discharged directly to psychiatric ward from burn care [16], potentially leading to overestimation of the length of injury related hospital stay. A third challenge is related to the quality of coding of diagnoses; it is known that E-coding of injury records may have been incomplete [17] and codes may be erroneously recorded in the register [7].

Although some of these challenges can be overcome if the data can be linked using personal identity codes such as in the Nordic countries including Finland, there is obviously a need for a transparent approach to addressing, clarifying and quantifying the key methodological issues and usability of hospital discharge data as a source for assessing fire-related injuries.

The purpose of this study was to examine methodological challenges, in particular issues concerning completeness of external cause coding, determination of incident cases and the amount of relevant inpatient days, in using the Finnish National Hospital Discharge Register (FHDR) for studying fire-related injuries. More specifically, the aims were to 1) provide suitable definitions for identifying fire-related discharge records and episodes from the FHDR, 2) investigate the completeness of recording of external causes of potentially fire-related injuries (burns and combustion gas poisonings) in the FHDR, and 3) study how different exclusion criteria for fire-related discharge records change the total amount of bed days.

## Methods

The Finnish national hospital discharge register (FHDR) was used as a data source in this study. The FHDR has a total (legislative) coverage of all inpatient care provided at university, general and mental hospitals or primary care health centres, as well as treatment in military and prison wards and private hospitals since 1969. Each record contains data on several variables, such as personal identity codes, age at admission, gender, hospital identifier code, admission and discharge dates, nature of injury (N-code), and external cause of injury (E-code). Each care record may contain multiple entries of N-codes; minimum number of fields for N-codes in the FHDR has been three. The first N-code indicates the main injury. Other N-codes are supplementary, indicating simultaneous injuries or conditions. The term ‘diagnosis’ used in the text refers to all N-codes entered and ‘main diagnosis’ to the first N-code. Coding in the FHDR was done according to the International Classification of Diseases and Related Health Problems -classification system’s 10^th^ version (ICD-10) [18] from 1996 onwards and according to the 9^th^ version [19] during the period 1987–1995. E-codes in use during the ICD-9 era were E880-E900 and during ICD-10 era diagnoses V01-Y89.

The quality of the FHDR has previously been considered generally good and it has been described as reliable and informative with good accuracy and completeness for epidemiologic studies [20-26]. General patterns and trends of E-code underreporting in the FHDR have also been examined and most severe problems were found to relate to the introduction of the ICD-10 classification shortly after 1996 [17]. After adaptation of ICD-10 underreporting of E-codes was also common for injuries other than burns [17], which suggests that the elevated proportion of unknown mechanism of injury is not exclusive to this study topic, but rather is an artifact of the introduction of the ICD-10 in Finland.

### Data selection and pre-processing

The cohort of patients selected for the purposes of this study were those having at least one discharge record in inpatient care with any of the E- or N-codes as explained in Table 1 in the FHDR during 1996–2009. By using the personal identity codes for the identified cohort of patients, all discharge records (including from day surgeries) from 1987–2009 were retrieved for these patients. During 1987–1995 records having an E-code indicating an injury caused by fire and flames (ICD-9: E890-E899) or a N-code indicating burns (ICD-9: 9400–9490) or combustion gas poisoning (ICD-9: 986) were assessed in order to monitor relevant care in the ICD-9 era.

**Table 1:** Fire-related injuries in discharge register and the explanations of the codes

**Explanations of the codes**	
ICD-10 code	Meaning
X00-X09	Exposure to smoke, fire and flames
X76	Intentional self-harm by smoke, fire and flames
X97	Assault by smoke, fire and flames
Y26	Exposure to smoke, fire and flames, undetermined intent
X47	Accidental poisoning by and exposure to other gases and vapours
T20-T32	Burns and corrosions
T58	Toxic effect of carbon monoxide
T59	Toxic effect of other gases, fumes and vapours

Coding associated with fire-related injuries according to the International Classification of Diseases and Related Health Problems -classification system’s 10^th ^version (ICD-10) and definition of records related to fire-related injuries.

Definition used for fire-related discharge records (any of the following three conditions is met):

• E-code in the set of {X00-X09, X76, X97, Y26}.

• E-code is X47 while any of the N-codes is within T20-T32.

• Any of the N-codes is within T20-T32 while simultaneously any of the other N-codes is within T58-T59.

Obtaining the complete histories of inpatient care for each person enabled the authors to form chains of care records by connecting chronologically subsequent inpatient records (referred to as care episodes from here on). A subsequent record was defined to belong to the same care episode if the preceding record’s discharge date was at maximum two days earlier than the following record’s admission date. Thus, transfers between hospitals, wards or between fields of specialised healthcare that each result in a separate record in the FHDR could be captured enabling definition of the complete inpatient time for each episode, regardless of possible missing or ambiguous coding in some of the records. If there was only a single record not chronologically connected as depicted above it was considered as a care episode described by only one record.

Also backward times from later burn episodes to previous burn episodes were investigated. Estimate for minimum clearance period required to exclude obvious readmissions was obtained using the method based on smoothed hazard function on backward time scale [27]. The basic idea was to detect how long the probability for a new admission due to same reason remains clearly elevated at the population level.

### Associating care episodes with mechanism of injury

**Figure 1:**
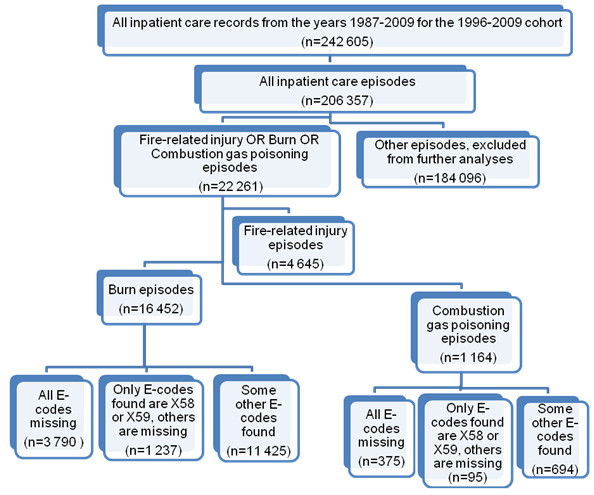
**Care episodes associated with corresponding mechanism (fire-related, burns, combustion gas poisonings). **E-codes X58-X59 represent accidental exposure to other and unspecified factors.

Classifying the care episodes into groups associated with mechanisms of injury, as depicted in Figure 1, serves the purpose of monitoring the completeness of E-coding among the types of injuries most commonly involved in fire-related injuries together with the care episodes associated with fire-related injuries by definition (Table 1). To be more specific, the burn and combustion gas poisoning episodes represent some potential to have been caused by any kind of injury including fire-related injuries. Monitoring these separate mechanism of injury groups together in time allowed the authors to infer from the mechanism identification issues induced by incompleteness in E-coding and how these issues progressed together over time.

### Completeness of E-coding

Inspecting the completeness of E-coding among single discharge records provides the most detailed and uncompromising picture on the quality of coding in the FHDR. The completeness was assessed among the two key diagnosis groups usually occurring among fire-related injuries, burns (ICD-10: T20-T32) and combustion gas poisonings (ICD-10: T58-T59) as well as among the type of care provider (university hospitals, central or district hospitals and health centres or private wards).

### Details of the ethical approval

The study protocol was approved by the Institutional Review Committee of National Institute for Health and Welfare (1/2011: §279/2011, 27.01.2011). Informed consent was not required since the data were anonymous register data and the people were not contacted.

## Results

### Records and care episodes

Retrieval of discharge records yielded in total 242 605 records which were classified as care episodes according to Figure 1. Table 2 reveals the numbers of care records and care episodes corresponding to each mechanism of injury. Of the obtained fire-related, burn and combustion gas episodes, 21% were fire-related (Table 1 and ICD-9: E890-899), 74% had a record with burn injury diagnosis (ICD-10: T20-T32, T95, ICD-9: 9400–9490) with no fire-related record and 5% had a record with combustion gas poisoning diagnosis (ICD-10: T58-T59, ICD-9: 986) with neither burn injury nor fire-related record present. Of the fire-related episodes, 81% contained a record of burn injury, 13% a record of combustion gas poisoning but no burn injury and the rest 6% records with other N-codes.

**Table 2:** Numbers of care records and care episodes retrieved from the Finnish National Hospital Discharge Register

	**Care records**	**Care episodes**
Total	242 605	206 357
Fire-related	5 736	4 645
Burn care	20 138	16 452
Combustion gas poisoning	1 317	1 164
Other type of care	215 414	184 096

### Patterns of E-coding among care episodes

**Figure 2:**
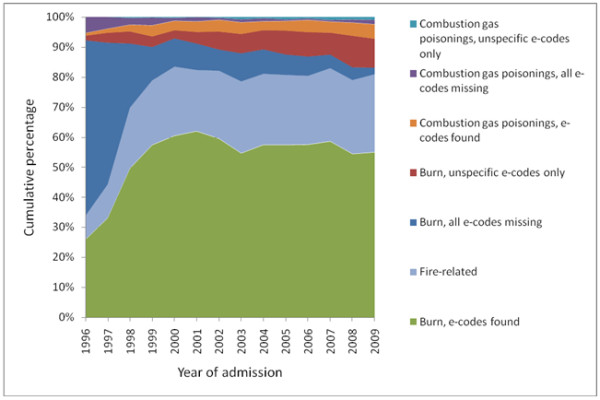
**Percentages of care episodes classified according to completeness of e-coding. **E-code completeness of potentially fire-related episodes displayed together with identified fire-related care episodes by year of admission.

The proportion of unknown causing mechanism for injury, that is, care episodes where all E-codes are missing, in both burn and combustion gas poisoning cases was strikingly elevated in the first few years after the implementation of ICD-10 (1996). However, it improved dramatically thereafter (Figure 2). The proportion of unspecific cause coding, that is, the only existing E-codes are X58 or X59 (accidental exposure to other and unspecified factors), has shown some increase during the last 10 years.

When inspecting the care episodes consisting of two or more discharge records it was observed that episodes may well contain records with both missing and present E-codes. For example, 17% (n = 381) of episodes of burn mechanism with some records having E-codes present (n = 2281) simultaneously included records that had a main diagnosis of burns (ICD-10: T20-T32, T95) but had missing E-codes.

### Completeness of external cause coding among single records

In 1996 about 2 out of 3 records with burns as the main diagnosis were missing E-codes while in 2009 the proportion was merely 2.3%. In the period 2000–2009 there has been a clear increase in usage of unspecific E-codes (X58, X59). The pattern is very similar for records with combustion gas poisoning as the main diagnosis (Table 3).

**Table 3:** The completeness of E-coding among burn and combustion gas poisoning records

	**Burns**			**Combustion gas poisonings**		
**Year**	**n**	**Unspecific (%)**	**Missing (%)**	**n**	**Unspecific (%)**	**Missing (%)**
1996	1813	1.9	65.6	126	0	77
1997	1834	4.1	53.3	104	0	66
1998	1675	5.6	25.5	110	5	26
1999	1664	4.2	12.6	143	2	27
2000	1749	3.7	10.9	107	5	14
2001	1693	4.7	10.2	104	1	19
2002	1577	6.4	8.6	111	5	8
2003	1603	8.3	10.5	132	12	11
2004	1577	7.9	8.6	130	6	11
2005	1586	9.8	7.3	110	14	7
2006	1559	9.6	7.9	132	5	5
2007	1375	9.8	3.9	135	10	4
2008	1543	13.6	4.5	177	9	5
2009	1429	11.4	2.3	185	11	10

Proportions (%) of unspecific (ICD-10: X58-X59) and missing E-codes in the single discharge records with burns (ICD-10: T20-T32) and combustion gas poisoning (ICD-10: T58-T59) as the main diagnosis and number of records (n) by year of admission.

The percentages of missing and unspecific E-codes were examined according to the type of care provider. From this perspective the completeness of coding was lowest in records originating from district/central hospitals, as shown in Table 4. During 2007–2009 the percentages of missing E-codes were 5% for health centre and private wards, 5% for district/central hospitals and 2% for university hospital type of care when considering the records with a burn diagnosis. The corresponding figures were 3%, 12% and 2% for records with a combustion gas poisoning diagnosis.

**Table 4:** The completeness of E-coding among care provider types

	**Combustion gas poisonings**					
	**E-code unspecific**			**E-code missing**		
	**Central or district hospital (n/%)**	**Health centre and private wards (n/%)**	**Universityhospital (n/%)**	**Central or district hospital (n/%)**	**Health centre and private wards (n/%)**	**University hospital (n/%)**
1996-1998	139/3	99/1	102/0	139/53	99/66	102/55
2007-2009	233/8	103/2	161/18	233/12	103/3	161/2
**Burns**						
1996-1998	2218/2	1181/0	1923/8	2218/43	§1181/64	1923/46
2007-2009	1741/16	767/7	1839/10	1741/5	767/5	1839/2

Proportion (%) of unspecific (ICD-10: X58-X59) and missing E-codes in single discharge records with burns (ICD-10: T20-T32) or combustion gas poisoning (ICD-10: T58-T59) as the main diagnosis and the total amount of cases (n) by type of care provider and year of admission.

### Detected problems in determining the true incidence of injury

**Figure 3:**
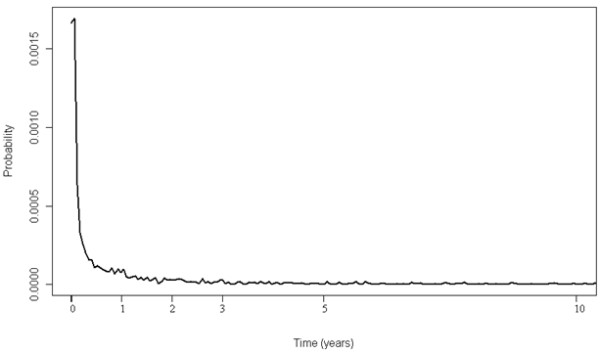
**Smoothed hazard function of previous burn episode. **Displays smoothed hazard as a function of time counted backwards (years) since previous burn episode.

Three types of challenges were encountered in defining the first admission for injury and therefore the true incidence of injuries:

1. Readmissions. Patients that have had multiple care episodes which contain a burn injury diagnosis exist. This is problematic if we are interested in the incidence of new injuries and not in the resource use as most of the new admissions are likely to be readmissions due to the earlier injury. Sequelae of burns do have a specific N-code (T95) in the ICD-10 classification system, but the extent of usage of this code instead of ‘acute’ codes (T20-T32) in cases of actual readmissions was not clear. The probability for a new episode remained elevated until a time of approximately 1-2-years after which it becomes nearly constant (Figure 3), i.e. at least two years clearance period should be used to exclude most obvious readmissions (capturing 91% of all readmissions within ten years), if only limited backward data are available to detect true first admissions.

2. Stability of injury mechanism. Finding the first admission for a fire-related injury naturally requires recognition of individuals in the data who experienced such injuries in the first place. When looking at all fire-related care episodes containing more than one care record from 1996–2009, annually 3 to 17 (2%–5%) of all fire-related care episodes contained potentially contradicting care records (ICD-10: X10-X19: Contact with heat and hot substances, X30-X39: Exposure to forces of nature, W32-W40: Some firearm and explosion injuries, W85-W99: Exposure to electric current, radiation and extreme ambient air temperature and pressure) in terms of the causing mechanism. This is problematic as the information obtained from several records belonging to the same episode may give different information on the injury mechanism and complicate the classification of injuries. For example injury from cooking oil on fire could possibly be E-coded as X12: contact with other hot fluids or X09: Exposure to unspecified smoke, fire and flames. Without knowing the causing mechanism this kind of coding could arise doubts whether one of the codes is invalid.

3. Injuries of institutionalised patients. It is possible for patients to have fire-related injuries while already being admitted to inpatient care, which may complicate defining the time of the injury. In the period 1996–2009, for 108 patients (3% of all patients) the first care record in their very first fire-related care episode did not indicate a fire-related E-code (ICD-10: X00-X09, X76, X97, Y26) or any diagnosis of burn injury (ICD-10: T20-T32, T95) or any diagnosis of combustion gas poisoning (ICD-10: T58-T59). In half of such first records the main diagnosis code belonged to the F-category (ICD-10: Mental and behavioral disorders) and in 32% the field of specialty was psychiatric care. The rest of these records possessed a variety of diagnosis codes from different categories of the ICD-10 classification system.

### Ambiguity in defining the total inpatient days

The impact of some intuitively attractive inclusion criteria for counting total inpatient days from the fire-related care episodes were analysed. The criteria were: A) care in the field of psychiatry excluded, B) care with a long-term care decision excluded, C) only care with an injury diagnosis in the three first N-codes (ICD-10: S00-T98) included, D) only care with an injury diagnosis as the main diagnosis included, E) only care with an E-code from X00-X09, X76, X97, Y26 included. This was performed by comparing the percentages of yearly total inpatient days obtained using inclusion criteria against the yearly total inpatient days of full, criterion free, inpatient time, as shown in Table 5.

**Table 5:** Impact of various inclusion criteria in counting inpatient days

**Median length of episode**	**2000**	**2001**	**2002**	**2003**	**2004**	**2005**	**2006**	**2007**	**2008**	**2009**
	**6**	**5**	**6**	**5**	**4**	**5**	**5**	**4**	**5**	**4**
A	54%	78%	89%	88%	84%	90%	78%	83%	91%	93%
B	52%	76%	98%	97%	79%	84%	86%	82%	91%	95%
C	52%	73%	86%	85%	69%	78%	76%	76%	80%	83%
D	48%	69%	83%	76%	67%	74%	70%	71%	76%	82%
E	42%	54%	68%	61%	55%	60%	61%	62%	67%	71%

Percentages (%) of total inpatient days when using limiting criteria in relation to the overall total of inpatient stays without limitation (100%) for fire-related care episodes, according to year. Limitation criteria: A) care in the field of psychiatry excluded, B) care with a long-term care decision excluded, C) only care with an injury diagnosis in the three first N-codes (ICD-10: S00-T98) included, D) only care with an injury diagnosis as the main diagnosis included, E) only care with an E-code from X00-X09, X76, X97, Y26 included.

Including all but the care in the field of psychiatry or including all but long-term patients usually led to a smaller difference when compared to the unlimited total than the other limitation criteria. The differences between these limitation criteria ranged from 2% to 48%.

When only inpatient care where the main diagnosis belonged to the category S00-T98 (ICD-10: Injury, poisoning and certain other consequences of external causes) were included the differences ranged from 17% to 52%. When this criterion was relaxed slightly so that it was enough for any of the three N-codes to belong to the category S00-T98 the difference was somewhat smaller, ranging from 14% to 48%.

The differences were clearly largest when only inpatient care with E-codes referring to fire-related injury was included. In this case they ranged from 29% to 58%.

## Discussion

In this study some methodological challenges in assessing fire-related injuries using Finnish National Hospital Discharge Register (FHDR) were examined. Rigorous analysis has demonstrated and clarified the most important issues and their potential impact on the feasibility of the FHDR as research data for assessing injuries caused by exposure to smoke, fire and flames.

To the authors knowledge, previous studies on this specific topic, aside from some burn injury studies in general [28-30] and a high quality study on methodological issues using the FHDR for hip fracture monitoring [27], do not exist.

The completeness of E-codes for burn injuries showed a dramatic improvement over time, although hindered by an increased use of unspecific codes. The use of unspecific E-codes provides little information regarding the injury, except that it was an injury caused by some external factor. Classification of causing mechanisms using information provided by whole care episodes, instead of a single record, is less sensitive to missing and unspecific E-codes as record-wise inspection would postulate and therefore a more reasonable approach. In some cases the episode may contain records with both missing and non-missing E-codes while allowing identification of the probable mechanism of injury. However, it would still be important, for the sake of research targeting the prevention of injuries, to include the E-code at least within some wider category of appropriate mechanisms, rather than to leaving it out completely, or to include more than only the unspecific code.

Determining the incidence of injuries turned out to require some caution arising from using secondary data as research data. In this study similar types of challenges to those posed to Sund [27] in his study on methodological issues using the FHDR for hip fracture monitoring were encountered. The FHDR does not contain any key variable that indicates the first admission for the injury.

In order to determine the incidence of fire-related injuries a decision must be made on whether some clearance time should be used to ascertain which of the care episodes refer to a new injury or if only the patient’s very first period should be used. Using a clearance period may seem an arbitrary way to proceed, as it is then necessary to decide the length of that period. Depending on the clearance period some re-admissions for old injuries could be assigned as true new injuries. Clearly, using only the very first period to identify incident cases is a more conservative way to proceed in terms of estimated incidence. If only the very first period is used then logically there is an assumption that consequent care episodes after the first one in the patient’s care history are related to that same injury. In terms of costs or resource usage this would be, on the contrary, a more liberal way to proceed in the event that a truly new injury had actually occurred after the first admission. To examine this matter a hazard function for assessing the hazard of previous burn care as a function of time elapsed since the previous care episode was produced. It was observed that a reasonable minimum clearance time for assigning an admission as a care episode for a new injury could be somewhere around two years.

A decision needs to be made on which care episode should be used for determining the mechanism, or whether or not a patient ought to be included at all. In the case of patients having care periods for which the records contain potentially contradicting mechanisms the issue is to decide if such a patient should be included as a person injured by smoke, fire or flames at all. Bearing in mind that the N- and E-codes are entered by hospital personnel during their work it is reasonable to assume occasional coding errors do happen. It should also be realised that when the perspective is more focused on the events that led to injury (i.e. the nature of the injury) rather than the nature of injuries per se, more ambiguity will necessarily be involved in the process. When envisaging the range of events that may cause burn injuries it becomes apparent that situations may arise where using a pre-determined, rather large set of codes (as ICD-10 is) becomes at least somewhat ambiguous. In such situations ‘the right’ code may not exist, but the interpretation is subjective. For example, should being burned by an electric arc while clothing is also set on fire by it be coded by X06 (ICD-10: Exposure to ignition or melting of other clothing and apparel), W87 (ICD-10: Exposure to unspecified electric current) or possibly by some entirely different code? Considering the nature of the whole process of a person being first injured then admitted to care - which may take place in several wards or hospitals, with each transfer between wards and hospitals yielding its own record in the register - it may be reasonable to proceed in a forgiving way by not excluding the cases with these kinds of potentially contradicting mechanisms recorded. In fact, differing codes may well describe the phenomenon more richly than just a single code. In fact, it could be beneficial that several E-codes could be assigned for each record, but that is not currently the case in the FHDR.

Assuming the admission date of the first care period will define the time of injury seems logical if the injury did not happen during the stay in hospital. If the first record in a care episode indicates care for an injury it is then intuitive to assume that the injury did not occur during care even though it is possible that a further injury may occur during care. In fact, it turned out to be more problematic to determine events that may plausibly have happened during care. The data showed that for many injury cases that are quite likely to have occurred while being admitted to inpatient care the preceding care was psychiatric inpatient care. Some external research results [16] support this.

This study illustrates that while there may be many kinds of intuitive criteria for inclusion of inpatient days caused by the injury there are also non-negligible differences between the outcomes of these choices. What seems to be quite apparent is that using only the relevant E-codes as limitation criteria results in a serious underestimation of the probable inpatient stay that should be ascribed to a particular injury event. It is also apparent that the exclusion of psychiatric and long-term care has an effect on this outcome. As previously stated, on many occasions where injury has occurred during inpatient care it is during psychiatric care that such incidences have taken place. On this basis it is reasonable to exclude psychiatric care, and possibly care with an indicator of a long-term care decision, in order to avoid overestimation of the inpatient stay due to seemingly unrelated care.

It must be noted that some of the methodological challenges discussed here may be specific to Finnish data as hospital discharge registers vary by country. For example, most countries do not have personal identifiers in their data which is, as shown by the current study, a major shortcoming if no field identifying the first admission is recorded. On the other hand, for the countries having both personal identifiers a date of injury field enable more easy identification for the episodes of care.

## Conclusions

The FHDR covers almost the entire population, thus completely avoiding many of the issues in coverage or representativeness that arise with traditional probability samples or one center data. The FHDR also includes personal identification numbers in which are constant over time enabling complete, truly longitudinal, follow-up of care regardless of transfers between wards and hospitals and occasional outpatient time. These aspects make the FHDR a unique and very powerful data source for injury research, and allow the use of well defined injury episodes in addition to separate discharge records. This study, however, shows that relatively small changes in the definitions and used exclusion criteria may change the obtained statistics, such as incidence or total bed days, considerably. It was also shown that there is still room for improvement in the recording of E-codes in the FHDR and hospital personnel should be encouraged to continue to pay attention to E-coding.

Inspite of the demonstrated challenges in using the FHDR as research data the authors conclude it to be feasible and suitable for assessing fire-related injuries as long as potential problems are acknowledged and properly taken into account.

## Competing interests

The authors declare that they have no competing interests.

## Authors’ contributions

KH participated in the conception and design of the study, acquisition and analysis of the data and drafting the manuscript. PL and PML participated in the design of the study, interpretation of the data and revising the manuscript critically for intellectual content. RS participated in the coordination of the design of the study, critically revising and drafting the content and interpretation of the data and conceptualization of backward time analysis. All authors read and approved the final manuscript.

## Pre-publication history

The pre-publication history for this paper can be accessed here:

http://www.biomedcentral.com/1472-6947/13/36/prepub
